# High rates of diagnostic discordance and co‐pathology: Insights into PSP from the NACC dataset

**DOI:** 10.1002/alz.70248

**Published:** 2025-05-09

**Authors:** Tanav Popli, Subhamoy Pal, Allyson Gregoire, Jonathan M. Reader, Alexis Passamani, Kelly M. Bakulski, Arijit Bhaumik, Emile Pinarbasi, Henry Paulson, Amanda Cook Maher

**Affiliations:** ^1^ Michigan Alzheimer's Disease Research Center Ann Arbor Michigan USA; ^2^ Department of Neurology University of Michigan Ann Arbor Michigan USA; ^3^ VA Ann Arbor Healthcare System, Neurology Service Ann Arbor Michigan USA; ^4^ Department of Epidemiology University of Michigan, School of Public Health Ann Arbor Michigan USA; ^5^ Department of Pathology University of Michigan Ann Arbor Michigan USA; ^6^ Department of Psychiatry University of Michigan Ann Arbor Michigan USA

**Keywords:** Alzheimer's disease research center, clinical‐pathologic correlation, co‐neuropathology, disease heterogeneity, national Alzheimer's coordinating center, progressive supranuclear palsy

## Abstract

**INTRODUCTION:**

Clinical features associated with accurate clinico‐pathologic correlation within cognitive‐predominant progressive supranuclear palsy (PSP) presentations have not been extensively studied. We identified features affecting PSP diagnosis within the National Alzheimer's Coordinating Center (NACC) dataset, a cognitive/behavioral research database.

**METHODS:**

Autopsied NACC participants with clinical or neuropathologic PSP diagnoses were categorized by diagnostic modality (Clinical‐only, Neuropathology‐only, both). Group differences in clinical, demographic, neuropsychologic, and neuropathologic features were assessed.

**RESULTS:**

Only 38.8% of neuropathologically identified PSP had clinical PSP diagnosis while 63.5% of clinically identified PSP had PSP pathology. Neuropathology‐only cases had fewer motor symptoms and better processing speed/executive functioning than the Clinical‐Neuropathology group. Nearly 70% demonstrated co‐neuropathologies including Alzheimer‐type, Lewy body, and corticobasal degeneration pathologies.

**DISCUSSION:**

Within NACC, discordant clinical and neuropathologic PSP diagnoses highlight the heterogeneity of PSP presentation, possibly driven by reliance on “classic” *ante mortem* symptoms. Further studies should assess the impact of co‐pathology on clinical neurodegenerative disease presentations.

**Highlights:**

- A high rate of clinico‐pathologic PSP diagnostic discordance is seen within NACC.
- Cases without clinical PSP diagnosis had fewer classic motor and cognitive symptoms.
- Isolated PSP neuropathology is uncommon, with nearly 70% showing co‐neuropathology.

## 1 BACKGROUND

Progressive supranuclear palsy (PSP), a debilitating and fatal neurodegenerative disease, is increasingly recognized for its diverse clinical presentations, high rates of discrepant clinico‐pathologic correlation, and underdiagnosis during life.^1^, ^2^, ^3^ Historically, clinical diagnostic criteria for PSP focused on a classic motor phenotype, encompassing postural instability and falls, vertical supranuclear gaze palsy, and levodopa‐resistant parkinsonism, which is known as Richardson‐Steele variant (PSP‐RS).^4^, ^5^ Yet, prominent cognitive/behavioral impairments have long been recognized as part of PSP,^6^ with evidence to suggest that PSP subtypes other than PSP‐RS may account for the majority (ranging from 54% to 75%) of PSP presentations in large neuropathologically‐defined samples.^7^, ^8^, ^9^, ^10^ Beyond the “classic” cognitive profile of PSP, typified by prominent executive dysfunction and slowed processing speed,^11^, ^12^, ^13^ cognitive impairments in domains such as speech/language, social cognition, visuospatial skills, and autonomic function are increasingly reported,^14^, ^15^ and have led to revised clinical diagnostic criteria from the Movement Disorder Society (MDS).^16^ These criteria recognize eight different clinical subtypes to account for the broader range of PSP presentations including predominantly cognitive presentations, such as PSP with predominant frontal presentation and PSP with predominant speech/language disorder.^16^

Despite increasing recognition of cognitive‐predominant presentations of PSP, most prior studies evaluating the accuracy of clinical diagnostic criteria for PSP in neuropathologically defined cohorts focused on motor‐predominant phenotypes of PSP and evaluated the accuracy of clinico‐pathologic correlation against other motor‐predominant neurodegenerative disorders, such as Parkinson's disease.^9^, ^17^, ^18^, ^19^, ^20^, ^21^ In contrast, the spectrum of features in non‐classic, cognitive‐behavioral presentations of PSP that correlate with underlying PSP pathology, particularly in the context of a cognitive/behavioral referral setting, has not been extensively studied. Addressing this gap in knowledge has been hampered by the lack of large, neuropathologically defined collections of PSP within primarily cognitively‐presenting populations, such as those derived from cognitive/behavioral referral centers. A comprehensive understanding of these features is needed to improve clinicopathologic correlation among the variety of PSP and differentiate these from other potentially overlapping presenting conditions, such as Alzheimer's disease or Lewy body dementia.

The National Alzheimer's Coordinating Center (NACC) dataset presents an ideal opportunity to investigate cognitive‐predominant presentations of PSP in a large collection of neuropathologically defined individuals in a cognitive/behavioral referral setting. Containing longitudinally collected data (the Uniform Data Set [UDS]) from 34 NIH‐funded Alzheimer's Disease Research Centers (ADRCs) across the United States, the NACC dataset is one of the largest longitudinal cohorts containing clinical, behavioral, genetic, and neuropathologic research data.^22^, ^23^, ^24^ The NACC has primarily focused on investigation of Alzheimer's disease, resulting in an extensive dataset derived from cognitive/behavioral referral settings. This offers a valuable and underutilized opportunity to investigate cognitive presentations of non‐Alzheimer's pathologies, such as PSP, which may aid in more accurate *ante mortem* clinico‐pathologic correlation and explore the contribution of co‐occurring neuropathologies such as Alzheimer's disease and related dementias.

In this observational analysis, we use the NACC dataset to investigate PSP within a large cognitive/behavioral referral population, basing our analysis on three groups: those who (1) received a clinical research diagnosis of PSP during life and had PSP neuropathology at the time of autopsy (Clinical‐Neuropathology group), (2) received a non‐PSP clinical research diagnosis during life but showed PSP neuropathology at autopsy (Neuropathology‐only group), and (3) received a clinical research diagnosis of PSP during life but did not show PSP neuropathology at autopsy (Clinical‐only group). We assessed differences in demographic, clinical, and neuropsychologic findings among these groups, as well as rates of concurrent pathologic findings (co‐pathology) to determine how PSP and other neuropathologies interface with clinical and neuropsychologic characteristics in the NACC dataset.

**RESEARCH‐IN‐CONTEXT**

1. **Systematic Review**: We reviewed literature regarding cognitive‐predominant presentations of progressive supranuclear palsy (PSP) in neuropathologically defined cohorts. The majority of autopsy‐defined PSP studies focused on differentiating *ante mortem* factors between movement‐predominant phenotypes with less focus on cognitive/behavioral presentations.
2. **Interpretation**: In a cognitive/behavioral referral database, reliance on “classic” *ante mortem* symptoms may drive high clinical‐neuropathologic discordance within PSP diagnosis and underestimate the heterogeneity of PSP presentation. High rates of co‐neuropathology in cognitive/behavioral PSP presentation may further contribute to high rates of diagnostic discrepancy.
3. **Future directions**: Future studies should investigate the role of co‐neuropathology on PSP clinical presentation and role of PSP neuropathology as a co‐pathology within other neurodegenerative diseases. There is future opportunity for National Alzheimer's Coordinating Center (NACC) and other large multicenter neuropathologically characterized datasets to optimize collected variables, enabling further insights into related neurodegenerative conditions, such as PSP.

## 2 METHODS

### 2.1 Study participants

**FIGURE 1:**
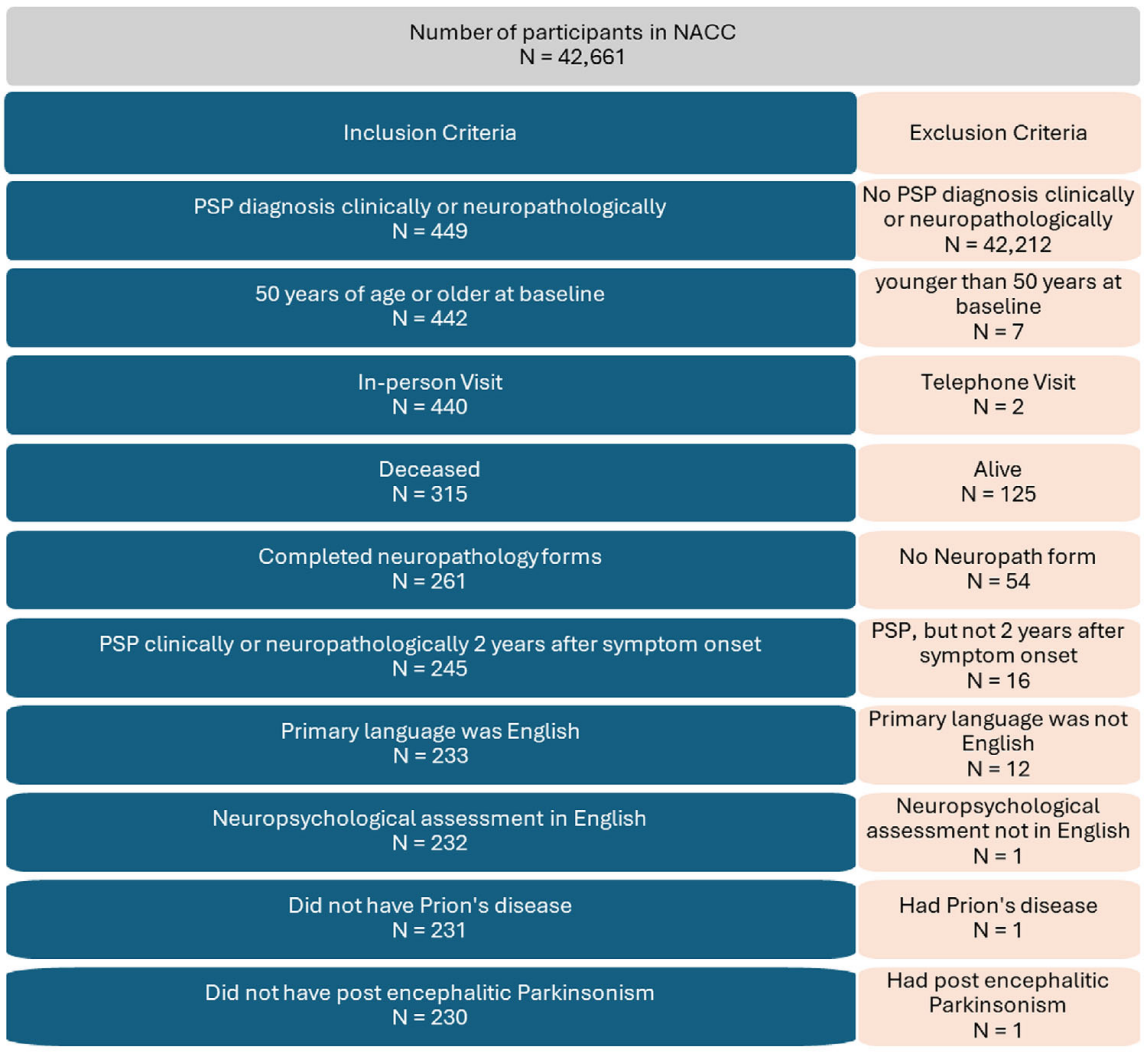
Study sample inclusion and exclusion criteria used to define the analytic cohort. NACC, National Alzheimer's Coordinating Center; PSP, progressive supranuclear palsy.

This study is a retrospective analysis of the NACC Uniform Data Set (UDS). Please see Figure 1 for details of the project criteria used to define the analytic cohort. In summary, at the time of project analyses, the UDS contained 150,744 participant visits between study initiation (2005) and March 2020 from 34 ADRCs across the United States. Of the 42,661 unique participants, 10,502 (24.6%) had died at the time of study analyses and of those, 6161 (58.7%) were autopsied. To be included in the present study analyses, participants had to have a clinical consensus diagnosis of PSP (primary or contributing) and/or neuropathologic evidence of PSP at autopsy. As specificity for clinic diagnosis of PSP peaks about 2 years from disease onset,^25^ clinical consensus diagnosis data were abstracted from the first in‐person study visit 2 years after reported symptom onset. If symptom onset occurred more than 2 years before a participant's first UDS visit, clinical consensus diagnosis from the UDS baseline (first) visit was used. Individuals without an age of symptom onset were excluded from analysis. To focus on PSP and minimize the potential contribution of co‐occurring conditions, participants younger than age 50 at baseline or diagnosed at autopsy with post‐encephalitic parkinsonism or prion disease were excluded. For interpretation of neuropsychological data, participants were excluded if their primary language was not English or if neuropsychological testing was not administered in English.

**FIGURE 2:**
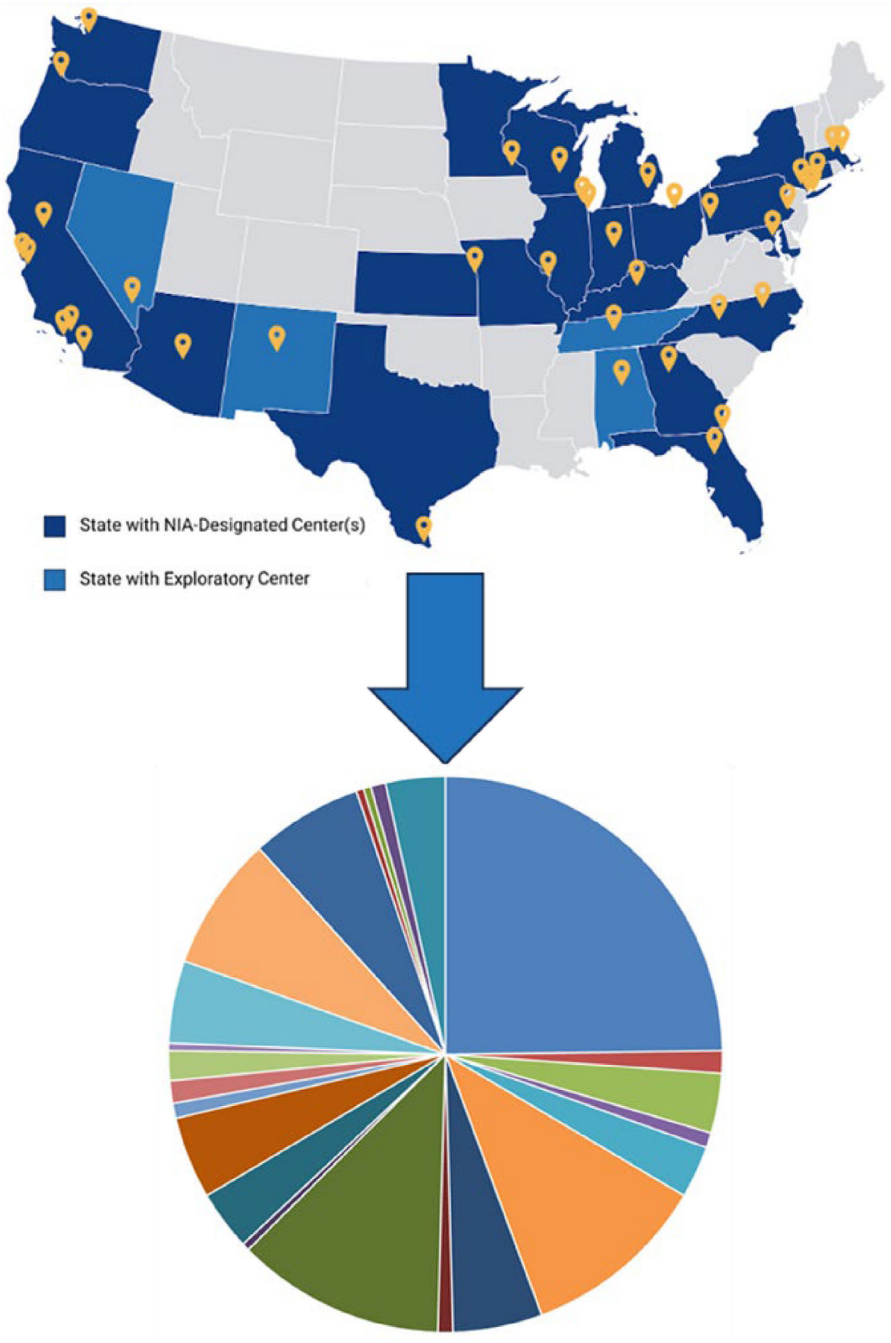
Cohort participants were drawn from 23 of the active ADRCs across the United States with no single ADRC providing the majority of participants. The map of the United States depicts all current ADRC locations across the country with states in blue having an ADRC (site indicated by the yellow dot). The pie chart depicts the relative contributions of single ADRCs to our cohort; with each color representing the proportion of participants from a different (although unspecified for confidentiality) ADRC. ADRC, Alzheimer's Disease Research Center.

The final sample consisted of 230 participants from 23 ADRCs (Figure 2): (1) 73 individuals clinically diagnosed with PSP as a primary or contributory etiology via NACC consensus from an in‐person study visit closest to 2 years after reported symptom onset and confirmed to have PSP neuropathology at autopsy (“Clinical‐Neuropathology” group), (2) 115 individuals diagnosed with PSP by neuropathology at autopsy but not clinically at their in‐person study visit closest to 2 years post symptom onset (“Neuropathology‐only” group), and (3) 42 individuals clinically diagnosed with PSP via NACC consensus at in‐person study visit closest to 2 years post symptom onset, but not neuropathologically at autopsy (“Clinical‐only” group).

### 2.2 Study measures

All participants completed baseline UDS procedures, including a clinician evaluation and neuropsychological assessment.^22^, ^24^ To obtain the largest possible sample, this study included individuals who completed UDS versions 1.2 (UDS1.2, implemented 03/2006,), 2 (UDS2, implemented 02/2008), or 3 (UDS3, implemented March 2015).

#### 2.2.1 Motor assessment

As part of the UDS clinician assessment, the presence or absence of motor symptoms was assessed. While there is some inconsistency in which motor symptoms were assessed across UDS versions, the presence/absence of gait disorder, falls, tremor, and slowness were measured across all three UDS versions. Here, we note the presence of these four motor symptoms on an item‐by‐item level and at a summary level (i.e., total number of motor symptoms present). As these symptoms were assessed at a study visit closest to 2 years post symptom onset, all motor symptoms noted in our cohort were necessarily present within the first 3 years of symptom onset, but are not inclusive of all early (within 3 years of onset) motor changes.

#### 2.2.2 Neuropsychological and behavioral measures^26^, ^27^

Collected neuropsychological and behavioral measures evolved over UDS versions. Previously published Crosswalk criteria^28^ were used to adjust UDS3 neuropsychological test scores to UDS1.2/2 equivalents. UDS3 neuropsychological measures without UDS1.2/2 equivalents, such as Benson Figure copy and delayed recall, are not included in the present analyses.

##### Mini‐Mental State Examination (MMSE)

The MMSE is a widely used, well‐validated screening measure for cognitive impairment. It measures orientation to time and place, immediate and delayed recall, calculation, language, and construction ability. The maximum total score is 30 points, with higher scores indicating better performance.

##### Digit Span

This test is comprised of two tasks: Digit Span Forward, a measure of immediate auditory attention span, and Digit Span Backward, a measure of verbal working memory. Total raw score is reported for each condition with higher scores indicating better performance.

##### Trail Making Test (TMT)

This is a test of visuomotor processing speed and executive functioning. For Part A of this test, individuals connect numbered circles in ascending numerical order as quickly as possible. For Part B, individuals connect circles alternating between numbers and letters in ascending numerical and alphabetical order as quickly as possible. Performance is measured in total seconds to complete the tasks, with lower scores indicating better performance. The tasks are discontinued after 150 and 300 seconds for Parts A and B respectively.

##### Boston Naming Test‐30 item (BNT‐30)

This is a test of visual object naming. The total number of correct responses recalled spontaneously or when given a semantic cue is recorded. Higher scores indicate better performance.

##### Semantic fluency (Animals, Vegetables)

Semantic fluency is a measure of speeded word retrieval to semantic cues. Individuals name as many items in a given category as they can within an allotted time. The number of unique responses named is scored and higher scores indicate better performance.

##### Phonemic fluency

Phonemic verbal fluency is a measure of speeded word retrieval to phonemic cues. Individuals name as many items that begin with a certain letter of the alphabet as they can in an allotted time. The number of unique responses is scored and higher scores indicate better performance.

##### Logical Memory

This test assesses the ability to recall a short story both immediately and after a delay. The number of accurately recalled story items is scored out of 25 and higher scores indicate better performance.

##### Geriatric Depression Scale, short form (GDS‐15)

This 15‐item questionnaire assesses symptoms of depression in older adults. Individuals answer yes or no to each question. Higher scores indicate greater levels of reported mood symptoms.

##### Neuropsychiatric Inventory (NPI‐Q)

This is an informant‐based interview that assesses dementia‐related behavioral symptoms. For each of 12 behavioral domains, an informant indicates whether symptoms have been present in the last month and, if so, symptom severity on a 3‐point Likert‐scale of severity (mild, moderate, severe). The 12 items are summed to make a total score, with higher scores reflecting greater neuropsychiatric burden.

Through NACC, participants complete annual assessments as long as they are willing and able. There are a number of reasons why participants may not complete all neuropsychological measures, including those related to disease symptom burden such as physical impairment (e.g., vision, motor difficulty) or cognitive impairment (e.g., language comprehension). Given the potential for non‐cognitive symptoms of PSP to impact a participants’ ability to complete neuropsychological testing, missing data for each neuropsychological test is reported.

### 2.3 Neuropathological characterization

Given the potential impact of co‐neuropathologies on clinical presentation, the presence of neuropathologies listed at the time of autopsy was abstracted from the NACC Neuropathology Data Form,^29^ including: Alzheimer's disease (AD), Lewy body pathology (LBD), frontotemporal lobar degeneration (FTLD‐tau, FTLD‐TDP) including non‐PSP FTLD‐tau subtypes (corticobasal degeneration, Pick's disease), and vascular pathologies (including cerebral amyloid angiopathy, infarcts and lacunes, microinfarcts, hemorrhages and microbleeds, and arteriolosclerosis). A category of “other pathological diagnosis” is included to capture the presence of co‐occurring neuropathologies such as argyrophilic grain disease‐limbic type (AGD), primary age‐related tauopathy (PART), and aging‐related tau astrogliopathy (ARTAG).

In the NACC dataset, individuals were determined to have FTLD‐tau (PSP) on a binary yes/no basis per NACC guidelines. Other FTLD neuropathology subtypes were similarly determined using a binary yes/no scale. Individuals were determined to have AD pathology according to commonly used pathologic criteria as specified by the NACC Neuropathology Data Form, such as National Institute on Aging (NIA)‐Alzheimer's Association guidelines or NIA/Reagan institute criteria: (1) Braak stage III‐VI and/or (2) moderate or frequent density of neocortical neuritic plaques (Consortium to Establish a Registry for Alzheimer's Disease [CERAD] C2 or C3). Lewy body pathology was determined on the basis of alpha‐synuclein staining (e.g., brainstem predominant limbic amygdala predominant, neocortical predominant, or unspecified). Vascular pathology was stratified by type, severity, and location as per NACC guidelines.

### 2.4 Statistical analyses

Descriptive statistics were generated for basic demographic and clinical characterization variables at the time of the in‐person UDS visit, using mean and standard deviation for numeric variables and count and frequency for categorical variables. We visualized the distribution of participants by ADRC site using a pie chart. First, we compared the Clinical‐only, Neuropathology‐only, and Clinical‐Neuropathology groups. Potential group differences were assessed using analysis of variance (ANOVA), chi‐squared, and Fisher's exact tests as appropriate. Tukey's post hoc analyses were applied to significant ANOVA tests to account for multiple comparisons. Holm corrections were applied to post hoc tests for significant chi‐squared and Fisher's exact tests to account for multiple comparisons. We visualized the comparison between clinical and neuropathology diagnoses using a Sankey plot.

ANOVA was used to assess potential group differences in neuropsychological test performance at the last in‐person visit. Tukey's post hoc analyses were applied for multiple comparisons. To correct for multiple comparisons within neuropsychological evaluation, Holm corrections were applied within cognitive domains (attention and working memory, processing speed and executive functioning, language, episodic memory, mood, and behavior). Potential group differences in missing neuropsychological data among the groups were analyzed using chi‐squared tests.

To evaluate whether the presence of neuropathologies in addition to PSP may explain differences in clinical PSP diagnosis, the presence of co‐neuropathologies was compared across groups using chi‐squared and Fisher's exact tests as appropriate. Holm corrections were applied to account for multiple comparisons. Tukey's post hoc analyses were applied for significant ANOVA test for multiple comparisons. We visualized the distributions of co‐pathologies using pie charts and bar charts.

## 3 RESULTS

**FIGURE 3:**
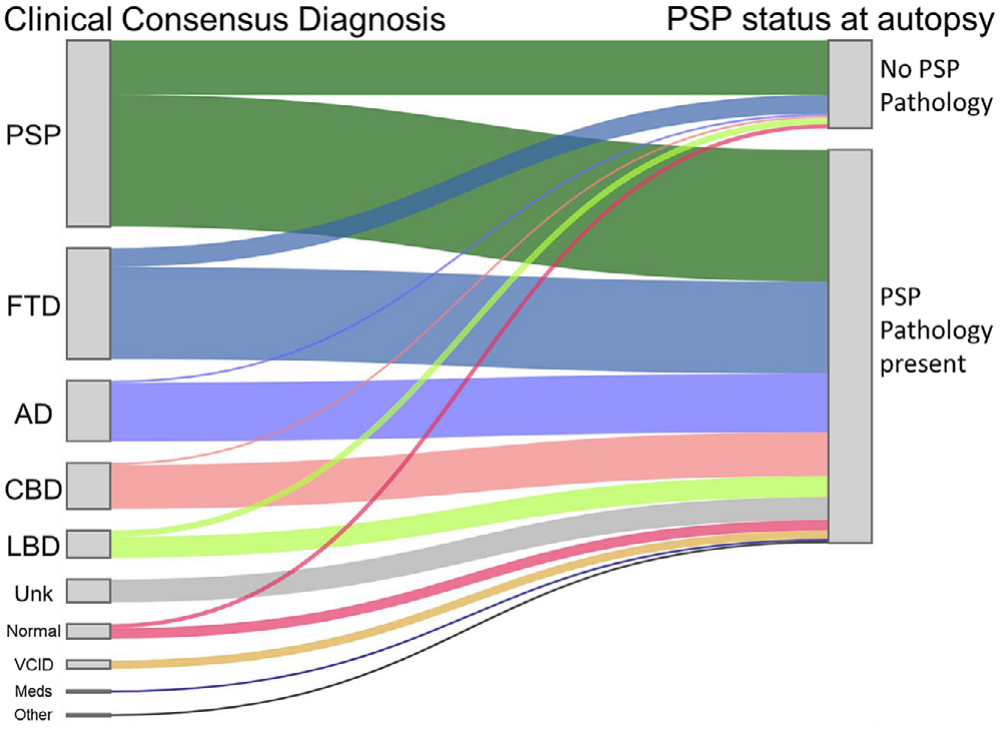
Comparison of primary clinical consensus diagnosis ∼2 years after symptom onset in relation to pathologic PSP status at autopsy. Width of the colored bars represents the number of individuals in that category. Individuals without a primary clinical consensus diagnosis of PSP and who did not have PSP pathology at autopsy had PSP as a contributing clinical diagnosis. AD, Alzheimer's disease; CBD, corticobasal degeneration; FTD, frontotemporal dementia; LBD, Lewy body disease; Meds, Cognitive impairment due to medication effects; Normal, normal cognition at consensus conference and no primary etiology recorded; Other, Cognitive impairment due to an unspecified other etiology; PSP, progressive supranuclear palsy; Unk, consensus conference diagnosis of “unknown”; VCID, Vascular cognitive impairment and dementia.

Of the 230 individuals included in study analyses, 73 (31.7%) were diagnosed with PSP both during life via clinical consensus and via neuropathology at autopsy (Clinical‐Neuropathology group). The remaining 157 (68.3%) participants had discordant clinical consensus and pathological diagnoses: 115 (50.0%) had Neuropathology‐only and 42 (18.3%) had Clinical‐only consensus diagnoses. Within the Neuropathology‐only group, the most common primary clinical consensus diagnoses in order of frequency were: frontotemporal dementia, Alzheimer's disease, corticobasal degeneration, and Lewy body disease, followed by non‐neurodegenerative etiologies (Figure 3.)

Demographic characteristics by group are shown in Table 1. The Neuropathology‐only group was significantly older than the other groups at their in‐person NACC visit and at death (*p*’s < 0.001). There was also a longer interval between the Neuropathology‐only group's NACC visit and time of death compared to the other groups (Clinical‐Neuropathology: *p* < 0.001; Clinical‐only: *p* = 0.04). While there was an overall statistical difference in the distribution of UDS versions completed by group, adjusted post hoc analyses did not demonstrate any significant between‐group differences. There were no other group demographic differences.

**TABLE 1:** Demographic and motor characterization of the study sample.

Characteristics	Overall sample (*n* = 230)	Clinical‐only PSP diagnosis (*n* = 42)	Neuropathology‐only PSP diagnosis (*n* = 115)	Clinical‐Neuropathology PSP diagnosis (*n* = 73)	Group difference *p*‐value	Post hoc adjusted *p*‐value
Age at visit Mean years (SD)	72.5 (8.9)	68.4 (9.0)	76.1 (8.6)	69.3 (7.0)	**<0.001** ^^^, ^*^	/
Tukey HSD post hoc		Neuropathology‐only versus Clinical‐Neuropathology Neuropathology‐only versus Clinical‐only				**<0.001** ^*^ **<0.001** ^*^
Age at death Mean years (SD)	75.8 (9.4)	71.2 (8.9)	79.9 (9.0)	71.8 (7.1)	**<0.001** ^*^	/
Tukey HSD post hoc		Neuropathology‐only versus Clinical‐Neuropathology Neuropathology‐only versus Clinical‐only				**<0.001** ^*^ **<0.001** ^*^
Time between visit and death Mean years (SD)	3.2 (2.4)	2.8 (2.6)	3.8 (2.6)	2.5 (1.8)	**<0.001** ^*^	/
Tukey HSD post hoc		Neuropathology‐only versus Clinical‐Neuropathology Neuropathology‐only versus Clinical‐only				**<0.001** ^*^ **0.04** ^*^
Female sex Number (%)	87 (37.8%)	14 (33.3%)	43 (37.4%)	30 (41.1%)	0.70	/
Race combined					^^^0.33	/
White	221 (97.4%)	41 (100%)	109 (95.6%)	71 (98.6%)		
Non‐White	6 (2.6%)	0 (2.4%)	5 (4.4%)	1 (1.4%)		
Unknown/ambiguous	3	1	1	1		
Ethnicity					^^^ 1.00	/
Non‐Hispanic	223 (97.4%)	41 (97.6%)	111 (97.4%)	71 (97.3%)		
Hispanic	6 (2.6%)	1 (2.4%)	3 (2.6%)	2 (2.7%)		
Unknown	1	0	1	0		
Education (years)	15.5 (2.9)	15.4 (2.9)	15.4 (2.9)	15.8 (3.0)	0.64	/
Missing	9	2	2	5		
Total number of motor symptoms present					**<0.001** ^^^, ^*^	/
Mean (SD)	2.2 (1.4)	2.8 (1)	1.5 (1.5)	3.1 (0.8)		
Unknown	1	0	1	0		
Tukey HSD post hoc		Neuropathology‐only versus Clinical‐Neuropathology Neuropathology‐only versus Clinical‐only				**<0.001** ^*^ **<0.001** ^*^
Gait disorder present Number (%)	158 (68.7%)	35 (83.3%)	53 (46.1%)	70 (97.2%)	**<0.001** ^*^	**/**
Chi‐squared adjusted post hoc		Neuropathology‐only versus Clinical‐Neuropathology Neuropathology‐only versus Clinical‐only Clinical‐Neuropath versus Clinical‐only				**<0.001** ^*^ **<0.001** ^*^ **0.01** ^*^
Falls present Number (%)	142 (61.7%)	29 (69%)	44 (38.3%)	69 (94.5%)	**<0.001** ^*^	**/**
Chi‐squared adjusted post hoc		Neuropathology‐only versus Clinical‐Neuropathology Neuropathology‐only vs Clinical‐only Clinical‐Neuropath versus Clinical‐only				**<0.001** ^*^ **0.001** ^*^ **<0.001** ^*^
Slowness present Number (%)	159 (69.1%)	38 (90.5%)	53 (46.1%)	68 (93.2%)	**<0.001** ^*^	**/**
Chi‐squared adjusted post hoc		Neuropathology‐only versus Clinical‐Neuropathology Neuropathology‐only versus Clinical‐only				**<0.001** ^*^ **<0.001** ^*^
Tremor present Number (%)	59 (25.8%)	14 (33.3%)	23 (20%)	22 (30.1%)	0.13	**/**
Not assessed	1	0	1	0		
Family history of neurodegenerative disease Number (%)	90 (39.13%)	15 (28.85%)	46 (40.0%)	29 (39.73%)	0.27	/
UDS form version					^^^ **0.02** ^*^	**/**
1	64 (27.8%)	7 (16.7%)	41 (35.7%)	16 (21.9%)		
2	144 (62.6%)	27 (64.3%)	65 (56.5%)	52 (71.2%)		
3	22 (9.6%)	8 (19%)	9 (7.8%)	5 (6.8%)		
Fisher's adjusted post hoc		*No significant differences*				0.069

*Note*: Data are presented for participants at their NACC visit that occurred closest to 2‐years post symptom onset. ANOVA was used to assess for mean group differences with use of Tukey HSD post hoc test to account for multiple comparisons. For categorical variables, chi‐squared or Fisher's exact tests were used to assess for group differences as appropriate with Holm corrections applied within post hoc tests to account for multiple comparisons.

* Significant group difference.

^^^ Use of Fisher's exact test.

Abbreviations: ANOVA, analysis of variance; HSD, honestly significant difference; PSP, progressive supranuclear palsy; UDS, Uniform Data Set.

Regarding assessed motor symptoms, the Neuropathology‐only group demonstrated significantly fewer overall motor symptoms compared to the groups with clinical consensus PSP diagnosis (*p *< 0.001). At the individual item level, a smaller proportion of the Neuropathology‐only group had gait disturbance, falls, and slowness present at their study visit closest to 2 years post symptom onset compared to the two groups with clinical consensus PSP diagnoses (*p*’s < 0.001). In addition, a significantly greater proportion of the Clinical‐Neuropathology group were rated as having a gait disorder and falls at the time of their in‐person UDS visit compared to the Clinical‐only group (*p*’s < 0.001).

Neuropsychological results from participants’ in‐person UDS evaluations are shown in Table 2, including the percentage of missing data for each test by group. Tests requiring upper extremity motor function (i.e., Trail Making Test) had the highest percentages of missing data, which is expected given PSP symptomatology. Group differences were also seen in the proportion of missing data for these tests, with the Neuropathology‐only group appearing to have fewer missing data than the Clinical‐only and Clinical‐Neuropathology groups (*p*’s < 0.05).

**TABLE 2:** Participant neuropsychiatric testing characteristics by group.

Characteristics	Overall sample (*n* = 230)	Clinical‐only PSP diagnosis (*n* = 42)	Neuropathology‐only PSP diagnosis (*n* = 115)	Clinical‐Neuropathology PSP diagnosis (*n* = 73)	*p*‐Value	Domain	Domain adjusted *p*‐value
Mini‐Mental State Examination Total Score (maximum: 30)	23.3 (6.1)	22.1(6.1)	23.2(6.2)	24.1 (6)	0.32	/	/
Missing	25 (11%)	7 (16.7%)	11 (9.6%)	7 (9.6%)	0.41		
Digit Span Forward Total raw score (maximum: 12)	6.9 (2.2)	6.6 (2.3)	6.9 (2.2)	7 (2)	0.68	Attention/ Working memory	/
Missing	49 (21.3%)	11 (26.2%)	20 (17.4%)	18 (24.7%)	0.34		
Digit Span Backward Total raw score (maximum: 10)	4.2 (2)	4 (2.4)	4.4 (2)	4.2 (2)	0.63	Attention/ Working memory	/
Missing	51 (22.2%)	12 (28.6%)	20 (17.4%)	19 (26%)	0.21		
Trail Making Test – A Time (sec) (maximum: 150)	79.4 (40.6)	86.3 (44.7)	70.1 (37.4)	95.6 (40.1)	**0.004** ^*^	Processing Speed/ Executive Functioning	**0.008** ^*^
Tukey post hoc			Clinical‐Neuropathology versus Neuropathology‐only				**0.004** ^*^
Missing	88 (38.3%)	19 (45.2%)	33 (28.7%)	36 (49.3%)	**0.011** ^*^		
Boston Naming Test‐30 Total raw score (maximum: 30)	23.7 (6)	23.3 (6.3)	23.5 (5.7)	24.1 (6.6)	0.82	Language	/
Missing	65 (28.3%)	15 (35.7%)	22 (19.1%)	28 ((38.4%)	**0.008** ^*^		
Animal fluency Total raw score (maximum: 31)	9.7 (5.7)	9.2 (5.4)	10.7 (5.9)	8.4 (5.2)	**0.033** ^*^	Language	0.099
			Clinical‐Neuropathology versus Neuropathology‐only				**0.03** ^*^
Missing	45 (19.6%)	13 (31%)	18 (15.7%)	14 (19.2%)	0.10		
Vegetable fluency Total raw score (maximum: 15)	6.4 (3.8)	6.4 (4.4)	6.9 (3.9)	5.6 (3.3)	0.17	Language	/
Missing	63 (27.4%)	15 (35.7%)	23 (20%)	25 (34.2%)	**0.042** ^*^		
Logical Memory IA Total raw score (maximum: 21)	7.5 (4.7)	7.8 (4.4)	7.3 (5)	7.7 (4.3)	0.85	Episodic memory	/
Missing	63 (27.4%)	15 (35.7%)	21 (18.3%)	27 (37%)	**0.008** ^*^		
Logical Memory IIA Total raw score (maximum: 21)	6.1 (4.7)	6.5 (4.6)	5.8 (4.9)	6.6 (4.4)	0.54	Episodic Memory	/
Missing	66 (28.7%)	14 (33.3%)	23 (20%)	29 (39.7%)	**0.011** ^*^		
Trail Making Test – B time (sec) (maximum: 300)	199.4 (82.6)	193.6 (80.2)	186.1 (81.9)	237.4 (76.3)	**0.02** ^*^	Processing Speed/ Executive Functioning	**0.04** ^*^
Tukey post hoc			Clinical‐Neuropathology versus Neuropathology‐only				**0.015** ^*^
Missing	118 (51.3%)	28 (66.7%)	44 (28.3%)	46 (63%)	**<0.001** ^*^		
Geriatric Depression Scale‐15 Total raw score (maximum: 14)	5 (3.9)	5.5 (4.2)	4.2 (3.7)	6 (3.7)	**0.011** ^*^	Mood/ Behavior	**0.022** ^*^
Tukey post hoc			Clinical‐Neuropathology versus Neuropathology‐only				**0.01** ^*^
Missing	50 (21.7%)	13 (31%)	21 (18.3%)	16 (21.9%)	0.23		
Neuropsychiatric Inventory‐Questionnaire Total raw score (maximum: 21)	6.5 (4.7)	7.6 (5.5)	5.4 (4.1)	7.6 (4.8)	**0.002** ^*^	Mood/ Behavior	**0.004** ^*^
Tukey post hoc			Neuropathology‐only versus Clinical‐only Neuropathology‐only versus Clinical‐Neuropathology				**0.02** ^*^ **0.004** ^*^
Missing	7 (3%)	0 (0%)	7 (6.1%)	0 (0%)	**0.029** ^^^, ^*^		

*Note*: Data are presented for participants at their NACC visit that occurred closest to 2‐years post symptom onset. ANOVA was used to assess for mean group differences. Holm corrections were applied to *p*‐values within cognitive domains to account for multiple comparisons and are shown in the “Domain Adjusted *p*‐value” column. For Missing Data analyses, chi‐squared tests were conducted to assess for potential group differences unless noted.

* Significant group difference.

^^^ Use of Fisher's exact test.

Abbreviations: ANOVA, analysis of variance; NACC, National Alzheimer's Coordinating Center; PSP, progressive supranuclear palsy.

With these missing data in mind, group differences were seen on measures of auditory working memory and visuomotor processing speed as well as in self‐reported depression symptoms (domain‐adjusted *p*’s < 0.05). Post hoc analyses demonstrate that the Neuropathology‐only group outperformed the Clinical‐Neuropathology group on measures of visuomotor processing speed and executive functioning (domain adjusted *p*’s = 0.008 and = 0.04, respectively). The Neuropathology‐only group also reported fewer depressive symptoms than the Clinical‐Neuropathology group (domain adjusted *p = *0.02) and fewer neuropsychiatric symptoms compared to both Clinical‐only and Clinical‐Neuropathology groups (domain adjusted *p* = 0.004).

**FIGURE 4:**
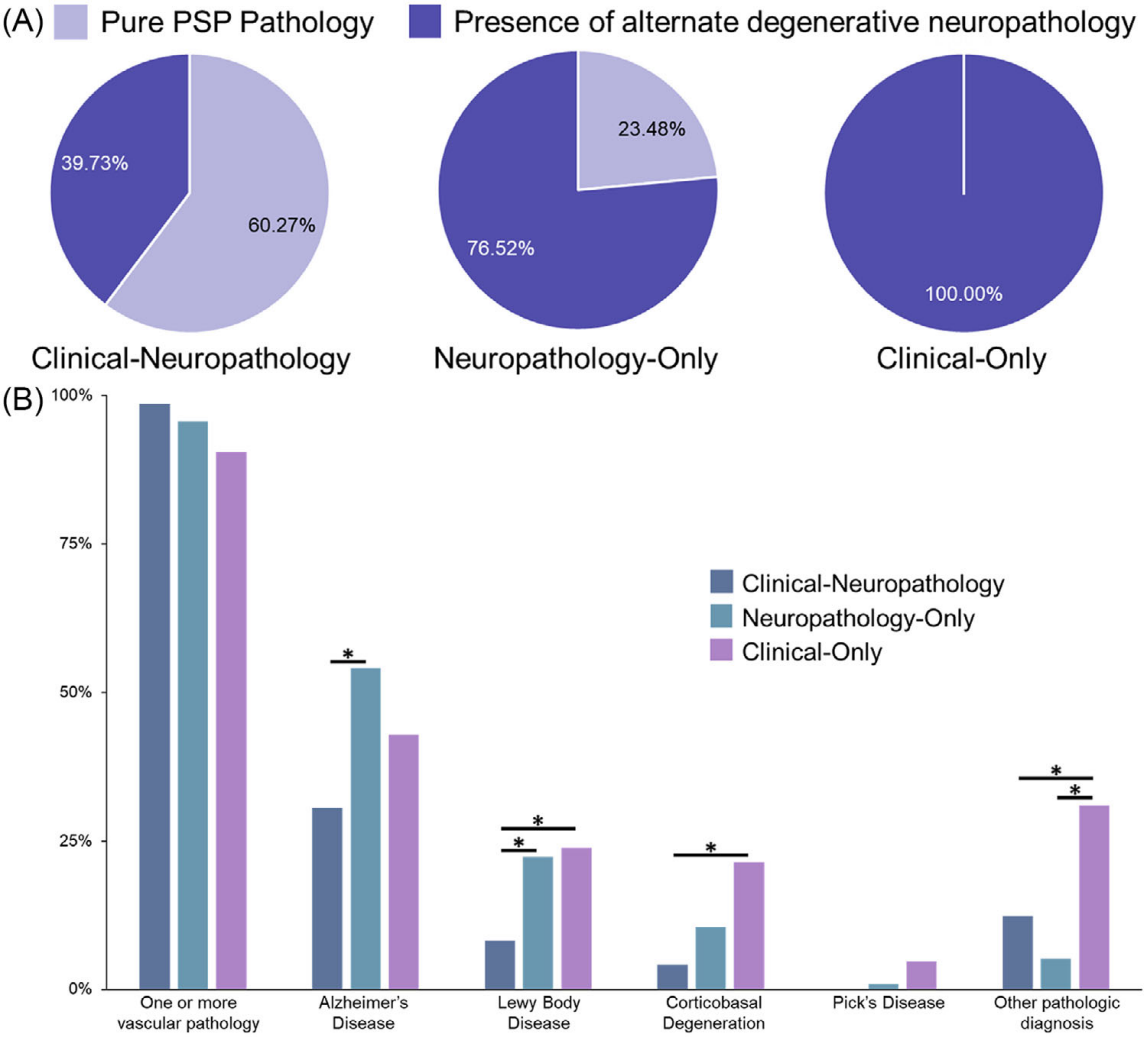
Co‐neuropathology is common in NACC‐dataset derived individuals with a neuropathologic or clinical diagnosis of PSP. (**A)** Rates of pure PSP pathology (lavender) and alternate neurodegenerative pathologies with or without concurrent PSP pathology (indigo) differed by clinic‐pathologic group. Pure PSP pathology was more common in participants with a concordant clinical consensus and neuropathologic diagnosis (Clinical‐Neuropathology group) while additional and alternate neurodegenerative neuropathologies were more common in groups with discordant clinical and neuropathologic diagnoses (Neuropathology‐only and Clinical‐only groups). (**B)** Rates of neuropathologic findings in addition to PSP pathology by clinico‐pathologic group. “Other pathologic diagnosis” refers to neuropathologic entities such as argyrophilic grain disease or aging‐related tau astrogliopathy. *Indicates significance between groups at a post hoc adjusted *p*‐value of less than 0.05. NACC, National Alzheimer's Coordinating Center; PSP, progressive supranuclear palsy.

Neuropathological co‐pathology findings for each study group are detailed numerically in Table 3 and visually represented in Figure 4. Overall, both vascular and neurodegenerative co‐neuropathology was common, demonstrated in 69.1% of the overall sample. Pure PSP neuropathology without other co‐neuropathologies (vascular or neurodegenerative) was seen in 30.9% of the study sample, although the rate of pure PSP was higher in the combined Clinical‐Neuropathology group compared to the Neuropathology‐only group (*p *< 0.001). The Clinical‐only group, by definition, did not contain any PSP pathology, and was more likely to harbor non‐PSP neurodegenerative pathologies compared to both the Clinical‐Neuropathology and Neuropathology‐only groups (*p*’s < 0.001). Nearly half of the overall study sample (44.4%) demonstrated Alzheimer's disease neuropathologic change (ADNC), although this was more commonly seen in the Neuropathology‐only group compared to the Clinical‐Neuropathology groups (*p = *0.005). Vascular co‐pathology was extremely common across the groups, particularly arteriolosclerosis; however, there were no significant differences in vascular co‐pathologies between groups. A smaller proportion of the study sample demonstrated Lewy body (LBD), corticobasal degeneration (CBD), and Pick's disease pathologies. The discordant diagnosis groups (Clinical‐only and Neuropathology‐only) had significantly greater proportions of individuals afflicted with LBD than the concordant Clinical‐Neuropathology group (*p *= 0.04). Individuals in the Neuropathology‐only group demonstrating LBD neuropathology tended to have a limbic or amygdala predominant pathology distribution relative to the Clinical‐only group (*p* = 0.02), while afflicted individuals in the Clinical‐only group had higher rates of neocortical Lewy body distribution relative to the Neuropathology‐only (*p* = 0.02) Similarly, the Clinical‐only group had a higher proportion of CBD pathology relative to the Clinical‐Neuropathology group (*p *= 0.02) and a higher proportion of “Other pathologic diagnoses” (representing neuropathologic entities such as ARTAG, AGD, and PART) than the Neuropathology‐only group (*p* < 0.001) and Clinical‐Neuropathology group (*p* = 0.03).

**TABLE 3:** Participant neuropathological features by group.

Pathology present	Overall sample (*n* = 230)	Clinical‐only PSP diagnosis (*n* = 42)	Neuropathology‐only PSP diagnosis (*n* = 115)	Clinical‐Neuropathology PSP diagnosis (*n* = 73)	*p‐*Value	Post hoc adjusted *p‐*value
Pure PSP	71 (30.9%)	N/A	27(23.5%)	44(60.3%)	**<0.001** ^*^	
Alzheimer's disease	100 (44.4%)	18 (42.9%)	60 (54.1%)	22 (30.6%)	**0.007** ^*^	
Chi‐squared adjusted post hoc		Clinical‐Neuropath versus Neuropathology‐only				**0.005** ^*^
Unknown/not assessed	5	0	4	1		
Lewy body disease	41 (18.1%)	10 (23.8%)	25 (22.3%)	6 (8.2%)	**0.03** ^*^	
Brainstem‐predominant	9 (4%)	3 (7.1%)	2 (1.8%)	4 (5.5%)	/	/
Limbic (transitional) or amygdala‐predominant	19 (8.4%)	1 (2.4%)	16 (14.3%)	2 (2.7%)	/	/
Neocortical (diffuse)	9 (4%)	6 (14.3%)	3 (2.7%)	0 (0%)		
Lewy bodies present, but region unspecified or found in the olfactory bulb	4 (1.8%)	0 (0%)	4 (3.6%)	0 (0%)		
Chi‐squared adjusted post hoc		Clinical‐Neuropathology versus Neuropathology‐only Clinical‐Neuropathology versus Clinical‐only				**0.04** ^*^ **0.04** ^*^
Unknown/not assessed	3	0	3	0		
Corticobasal degeneration	24 (10.5%)	9 (21.4%)	12 (10.5%)	3 (4.1%)	^^^ **0.02** ^*^	
Unknown/not assessed	1	0	1	0		
Fisher's adjusted post hoc		Both versus Clinical‐only				^^^ **0.02** ^*^
Pick's disease	3 (1.3%)	2 (4.8%)	1 (0.9%)	0 (0%)	0.12	
One or more vascular pathology	215 (95.6%)	38 (90.5%)	108 (95.6%)	69 (95.8%)	0.12	
Unknown/not assessed	5	0	2	3		
Cerebral amyloid angiopathy	76 (63.3%)	14 (33.3)	37 (32.7)	25 (36.2%)	0.89	
Mild	40 (17.9%)	8 (19%)	17 (15%)	15 (21.7%)		
Moderate	27 (12.1%)	5 (11.9%)	15 (13.3%)	7 (10.1%)		
Severe	9 (4%)	1 (2.4%)	5 (4.4%)	3 (4.3%)		
Unknown/not assessed	6	0	2	4		
Infarct and lacunes	31 (13.5%)	3 (7.1%)	20 (17.4%)	8 (11%)	^^^0.23	
Microinfarcts	38 (16.6%)	4 (9.5%)	23 (20%)	11 (15.3%)	^^^0.29	
Unknown/not assessed	1	0	0	1		
Hemorrhages and microbleeds	15 (7.1%)	1 (2.6%)	9 (8%)	5 (8.3%)	^^^0.55	
Unknown/not assessed	19	3	3	13		
Arteriolosclerosis	165 (80.5%)	37 (90.2%)	79 (79%)	49 (76.6%)	0.82	
Mild	90 (43.9%)	23 (56.1%)	35 (35%)	32 (50%)		
Moderate	51 (24.9%)	14 (34.1%)	28 (28%)	9 (14.1%)		
Severe	24 (11.7%)	0 (0%)	16 (16%)	8 (12.5%)		
Unknown/not assessed	25	1	15	9		
Other pathologic diagnosis	28 (12.2%)	13 (31%)	6 (5.2%)	9 (12.3%)	**<0.001** ^*^	
Chi‐squared adjusted post hoc		Clinical‐only versus Neuropathology‐only Clinical‐only versus Clinical‐Neuropath				**<0.001** ^*^ **0.03** ^*^

*Note*: Pure PSP was defined as the presence of PSP neuropathology in the absence of other neurodegenerative pathologies; vascular pathology was not included as a variable in the determination of pure PSP pathology. Chi‐squared or Fisher's exact tests were used to assess for group differences as appropriate. Holm corrections were applied to post hoc tests to account for multiple comparisons.

* Significant group difference;

^^^ Use of Fisher's exact test.

Abbreviations: PSP, progressive supranuclear palsy.

## 4 DISCUSSION

This project leveraged the data‐rich, longitudinal NACC database to characterize individuals diagnosed with PSP via clinical consensus conference, autopsy, or both within a large sample biased towards cognitive/behavioral presentations in order to understand (a) symptomatic *ante mortem* differences contributing to accurate clinico‐pathologic correlation of PSP among ADRCs and (b) neuropathological differences among the groups with regard to non‐PSP co‐neuropathology. Overall, we observed a high rate of discordance between NACC clinical consensus diagnosis and neuropathological findings of PSP: only 38.8% of neuropathologically‐confirmed PSP cases were identified via NACC clinical consensus as having PSP, and only 63.5% of NACC clinical‐consensus identified PSP cases were confirmed to have PSP neuropathology upon autopsy. This diagnostic discordance highlights the complexity of accurate clinico‐pathologic correlation in PSP and may be driven by a historical reliance on classically described PSP symptoms during life.

The Neuropathology‐only group had fewer motor and neuropsychiatric symptoms than both clinically identified PSP groups. They also had better neuropsychological test scores in visuomotor processing speed and executive functioning, domains typically associated with subcortical dementias, and fewer depressive symptoms than the Clinical‐Neuropathology group. Given these differences, a reliance on traditionally described PSP symptoms, comprising the Richardson‐Steele subtype (PSP‐RS),^10^ may explain the lack of clinical PSP diagnosis in the Neuropathology‐only group. Research suggests PSP‐RS accounts for only 24%–46% of cases where PSP neuropathology is identified on autopsy^7^, ^8^, ^9^, ^10^, ^30^ and thus over‐reliance on PSP‐RS symptoms may contribute to misclassification.

Increased awareness of PSP symptom heterogeneity led to revised clinical diagnostic criteria from the MDS which now recognizes eight subtypes.^16^ This expanded classification represents an important step toward inclusion of alternate PSP presentations but may still overrepresent PSP‐RS subtypes given continued emphasis on postural instability.^5^ Given the MDS criteria's relatively recent release, it has not been available for the longitudinal NACC dataset, making this a limitation to the present study. An update to the UDS (version 4) has recently been released and includes the Unified Parkinson's Disease Rating Scale as a standard component and a recommendation to give the PSP‐Rating Scale for participants with features of PSP or CBD, which may enable better quantification of PSP‐related symptoms and better capture the range of clinical features associated with PSP than is presently possible.

Individuals diagnosed with PSP via clinical consensus who lacked PSP neuropathology at autopsy are an interesting group. They demonstrated high rates of LBD and CBD neuropathologies, similar to prior autopsy studies.^31^ Both LBD and CBD are associated with clinical syndromes characterized by impaired motor function and frontal/subcortical neuropsychological impairments.^32^, ^33^ The overlapping clinical presentation of PSP and these syndromes increases the risk of discordant clinico‐pathologic diagnosis in the research setting, subsequently impacting potential enrollment and outcomes of clinical trials for emerging pathology‐directed therapeutics. Recent work using the NACC dataset employed machine learning to identify *ante mortem* clinical features predictive of underlying neuropathology.^34^ While Phongpreecha and colleagues did not include PSP neuropathology in their analyses, perhaps due to the relatively smaller number of PSP cases within the NACC database, Vaccaro and colleagues (2020) used machine learning to classify a cohort of individuals with PSP, idiopathic Parkinson's disease, or normal health based on neuropsychological test performance outside of the NACC cohort. Results of the latter study were encouraging, with high classification accuracy (> 86% across groups, and 95% accuracy for PSP).^35^ This type of approach may be useful in the future to determine which clinically assessed variables may be most predictive of underlying PSP neuropathology within the NACC cohort.

Across the study sample, both vascular and neurodegenerative co‐neuropathology was very common, adding to recent research demonstrating high rates of multiple coincident pathologies across neurodegenerative diseases.^36^, ^37^, ^38^, ^39^ Substantial vascular co‐neuropathology was seen among all three groups (≥90%), consistent with prior neuropathologic assessment within the NACC cohort, although NACC participants with AD have historically shown higher rates of some vascular neuropathologies compared to those with other neurodegenerative processes, including tauopathies.^40^, ^41^ Within NACC, vascular pathology alone does not account for the discrepancy between clinical and pathologic PSP diagnosis. ADNC was the most prevalent neurodegenerative co‐neuropathology across study groups, as expected given NACC's historic focus on AD and general increase in ADNC with age; however, ADNC has been reported as a common co‐pathology in other pathologically‐confirmed PSP cohorts,^42^, ^43^, ^44^ suggesting this finding may not be specific to the NACC PSP cohort.

Our observation that “pure” PSP neuropathology represented a minority within our sample, consistent with autopsy cohorts spanning broad presentations across neurodegenerative diseases, where up to 71% of pathologically diagnosed PSP cases had at least one neurodegenerative co‐neuropathology and 22% demonstrated multiple co‐neuropathologies,^42^, ^45^ implies a significantly higher complexity within the pathology of PSP than suggested by previous PSP‐only cohorts^46^ and underscores the necessity of considering the effect of these additional pathologies on PSP diagnosis.

We found discrepancy in co‐neuropathology prevalence by diagnostic group, with the Neuropathology‐only PSP group having substantially greater co‐neuropathology (76.5%) than the Clinical‐Neuropathology group (39.7%). ADNC was particularly prevalent in the Neuropathology‐only group compared to the concordant Clinical‐Neuropathology group, in line with a recent large autopsy study which found that comorbid severe AD was more common in individuals with PSP neuropathology at autopsy who never received a clinical diagnosis of PSP.^3^ We questioned whether higher rates of ADNC contributed to the lack of clinical consensus PSP diagnosis in the Neuropathology‐only group, perhaps mediated by atypical clinical symptom presentation; however, we found no group differences in memory or language performance. Nonetheless, research suggests tau pathology burden, not ADNC, correlates with cognitive impairment in PSP.^44^, ^47^ Additionally, the higher rates of ADNC, and co‐neuropathology in general, within the Neuropathology‐only group may reflect the significantly older age of this group at autopsy relative to the clinically defined groups, as the occurrence of co‐neuropathology in autopsy‐confirmed PSP cohorts is significantly associated with older age.^42^, ^44^ However, our findings that other age‐associated neuropathologies, such as LBD, were similarly prevalent in the older Neuropathology‐only group and younger Clinical‐only group, suggests that age alone does not account for all co‐neuropathology in our sample. Alternatively, the presence of PSP neuropathology without a clinical PSP diagnosis in one‐half of our cohort suggests that PSP may be a common secondary pathology coinciding with primary pathologic diagnosis of ADNC. This is consistent with recent studies demonstrating a higher pathologic incidence of PSP than previously recognized.^3^, ^48^, ^49^ The high frequency of co‐neuropathology noted in the present study aligns with the emerging recognition that multi‐proteinopathy is a common feature of age‐related degenerative diseases.^36^, ^37^, ^38^, ^39^, ^42^ While the tau accumulation of AD differs histopathologically and molecularly from tau accumulation in PSP, the coexistence of AD and PSP pathologies in some individuals could reflect parallel and coincident responses to stressors, for example oxidative stress or inflammatory processes, affecting distinct cell populations. Another possible underpinning of multi‐proteinopathy, such as coexisting LBD and PSP pathology, is cross‐seeding of fibrillar formation by amyloidogenic disease proteins.^50^ The degree to which PSP pathology contributes to clinical presentations or pathophysiologic mechanisms in mixed pathology cases remains poorly understood, and there may be additional protective or predisposing factors deserving of further study. Our results suggest that greater attention to the presence of co‐neuropathology and its potential impact on the development and clinical features of neurodegenerative diseases during life may be warranted within the NACC dataset.

The NACC dataset has many advantages given its large cohort of longitudinal data collected from across the United States that allowed us to identify a neuropathologically‐defined sample comparable in size to many previous PSP autopsy studies^2^, ^7^, ^9^, ^16^, ^31^, ^47^, ^51^; however, there are limitations to this type of dataset. (1) Large, multisite studies such as NACC cannot capture individualized clinical details that make smaller studies more “narratively rich.” For example, only the presence or absence of select motor symptoms were available in this study, not symptom severity. (2) Changes over time in collection methods may also hamper longitudinal analysis. In this study, we successfully used published Crosswalk criteria^28^ to merge UDS neuropsychological assessment versions 1.2/2 and 3 to allow cross‐version comparison of neuropsychological data; however, data not included in previous UDS versions, such as the presence or absence of abnormal eye movements, cannot be retrospectively collected from individuals after their death. Our cohort may underestimate clinical cases of PSP within NACC due to lack of specific clinical details or conflicting information within UDS. (3) Given NACC's historical focus on AD, more limited details have been collected regarding frontotemporal lobar degeneration presentations and pathologies. This is particularly true with regard to movement disorder symptoms, where there is a historic sparseness of data. For example, and as noted above, the NACC dataset has not historically included collection of the PSP‐Rating Scale or the Frontal Assessment Battery, both of which have been recommended for use by the MDS for assessment of global and frontal lobe function, respectively, in PSP.^52^, ^53^ Without standardized historical collection of symptoms such as oculomotor dysfunction, postural instability, akinesia, and frontal lobe function, we lack the data to make accurate clinical PSP subtype classifications retrospectively within the NACC dataset. The recently released UDS version 4 does contain the UPDRS as a standard measure and recommends use of the PSP‐Rating Scale in participants with features of PSP and CBD, which will greatly enhance quantification of motor symptoms of NACC participants and potentially enable future research to investigate clinico‐pathologic correlation across PSP subtypes within NACC. (4) The NACC neuropathology dataset lacks tau staging metrics, which has implications for PSP diagnosis and staging as differences in tau propagation and regional distribution may underlie differences in clinical phenotypes.^54^, ^55^ Additionally, a lack of detailed information on non‐AD pathologies makes retrospective determination of primary or secondary pathologic diagnosis challenging, particularly absent standardized neuropathologic methods across NACC and historic difficulties distinguishing PSP pathology from other neurodegenerative tauopathies.^56^ (5) Finally, despite NACC being a nationally distributed sample, it does not represent the racial/ethnic diversity or socioeconomic diversity of the United States, overrepresenting White and highly educated older adults. This limits the generalizability of study findings to the broader population.

In summary, we successfully used the longitudinal NACC database to investigate the non‐AD condition of PSP. Leveraging both NACC clinical consensus and autopsy‐confirmed PSP diagnoses, we identified a high rate of clinico‐pathologic discordance within the NACC sample, perhaps due to overreliance on classical PSP symptoms during life; this is an area of growth for future UDS versions to reduce potential for misdiagnosis in the research setting. Co‐neuropathology was high across PSP diagnostic groups, particularly vascular and AD co‐pathologies. While this may reflect generally high rates of multiple coincident pathologies in age‐related neurodegenerative diseases, disproportionately high co‐pathology rates in the Neuropathology‐only PSP group suggests co‐pathology may influence discordant clinico‐pathologic correlation or that PSP may be a more common secondary pathology in other neurodegenerative conditions than previously recognized. While this study has inherent limitations, it sets the stage for further work investigating cognitive‐ and behavioral‐predominant PSP subtypes in individuals with clinical and/or autopsy‐confirmed PSP diagnosis. The NACC's timely UDS4 updates that include standard administration of the UPDRS and recommendation for the PSPRS to be completed in specific circumstances will greatly aid future investigations aimed at understanding the factors contributing to accurate *ante mortem* clinico‐pathologic correlation in PSP, both within and beyond the ADRC network.

## CONFLICT OF INTEREST STATEMENT

The authors declare no conflicts of interest. Author disclosures are available in the Supporting Information.

## CONSENT STATEMENT

All human participants provide informed consent to have their coded data included in the National Alzheimer's Coordinating Center (NACC) research database.

## Supporting information



Supporting Information
